# Successive influenza virus infection and *Streptococcus pneumoniae *stimulation alter human dendritic cell function

**DOI:** 10.1186/1471-2334-11-201

**Published:** 2011-07-20

**Authors:** Yuet Wu, Huawei Mao, Man-To Ling, Kin-Hung Chow, Pak-Leung Ho, Wenwei Tu, Yu-Lung Lau

**Affiliations:** 1 Departments of Paediatrics & Adolescent Medicine, Li Ka Shing Faculty of Medicine, University of Hong Kong, Hong Kong SAR, China; 2 Departments of Microbiology, Li Ka Shing Faculty of Medicine, University of Hong Kong, Hong Kong SAR, China

## Abstract

**Background:**

Influenza virus is a major cause of respiratory disease worldwide and *Streptococcus pneumoniae *infection associated with influenza often leads to severe complications. Dendritic cells are key antigen presenting cells but its role in such co-infection is unclear.

**Methods:**

In this study, human monocyte derived-dentritic cells were either concurrently or successively challenged with the combination of live influenza virus and heat killed pneumococcus to mimic the viral pneumococcal infection. Dendritic cell viability, phenotypic maturation and cytokine production were then examined.

**Results:**

The challenge of influenza virus and pneumococcus altered dendritic cell functions dependent on the time interval between the successive challenge of influenza virus and pneumococcus, as well as the doses of pneumococcus. When dendritic cells were exposed to pneumococcus at 6 hr, but not 0 hr nor 24 hr after influenza virus infection, both virus and pneumococcus treated dendritic cells had greater cell apoptosis and expressed higher CD83 and CD86 than dendritic cells infected with influenza virus alone. Dendritic cells produced pro-inflammatory cytokines: TNF-α, IL-12 and IFN-γ synergistically to the successive viral and pneumococcal challenge. Whereas prior influenza virus infection suppressed the IL-10 response independent of the timing of the subsequent pneumococcal stimulation.

**Conclusions:**

Our results demonstrated that successive challenge of dendritic cells with influenza virus and pneumococcus resulted in synergistic up-regulation of pro-inflammatory cytokines with simultaneous down-regulation of anti-inflammatory cytokine, which may explain the immuno-pathogenesis of this important co-infection.

## Background

Influenza virus is a major cause of respiratory disease and bacterial infection associated with influenza often increases morbidity and mortality [1,2]. Viral and bacterial co-infection is a common cause of death in seasonal [3-8] and pandemic influenza [9-13]. *Streptococcus pneumoniae *is the major bacterial pathogen associated with influenza in 1918 Spanish pandemic [9,11-13]. In the 2009 H1N1 pandemic, 29% of fatal cases of influenza is reported to have evidence of bacterial co-infection, with *Streptococcus pneumoniae *is the predominant type of pathogen identified [10]. Given the clinical importance of this co-infection and the increasing risk of influenza outbreak, it is important to understand how the immune system would be affected in dealing with the dual infection of these two pathogens.

Various studies have investigated the possible mechanisms of the co-infection in inducing disease severity [14,15] and these can be classified into four broad categories, which are prior influenza infection would (i) cause epithelial damage, (ii) increase pneumococcal adherence, (iii) dysregulate immune system components and (iv) enhance inflammatory response, such that the effect of subsequent bacterial infection will be exacerbated.

Dendritic cells (DCs) are professional antigen presenting cells that are highly potent at initiating and regulating immune response to control infection. Co-infection could alter innate cell responses [16] and different populations of cell in the immune system have been studied in the co-infection model of influenza virus and pneumococcus [17-20]. Despite the critical role of DCs in immune regulation, the response of DCs to this important co-infection has not been reported yet. We hypothesize that co-infection may alter DCs functions, leading to the dysregulation of immune functions and the elevated inflammatory response.

Pneumococcus is a human specific pathogen, however most related studies on co-infection are conducted in mouse model. It is demonstrated that human and murine DCs differ in their inflammatory response to pneumococcus [21]. In light of this, we investigated the immune response of human monocyte derived-dendritic cells (MoDCs) to concurrent or successive challenge of live influenza virus and heat killed *S. pneumoniae *to mimic the co-infection. MoDC viability, phenotypic maturation and cytokine production were examined. We showed for the first time a time dependent pattern of human MoDC functions alteration in response to varying dose of secondary pneumococcal challenge after influenza virus infection, which is useful to help understand the pathogenesis of this co-infection. This time and dose related interaction of influenza virus and pneumococcus in enhancing MoDCs pro-inflammatory cytokines may be the first trigger for the enhanced lung inflammation in patients with co-infection.

## Methods

### In vitro generation of MoDCs

Human peripheral blood was obtained from healthy donors in accordance with an approved protocol from the Institutional Review Board of The University of Hong Kong/Hospital Authority Hong Kong West Cluster. Peripheral blood mononuclear cells (PBMCs) were isolated from whole blood samples by Ficoll-Paque Plus (GE Healthcare Life Science) gradient centrifugation [22]. Monocytes were magnetically separated from PBMCs by positive selection using anti-human CD14 conjugated magnetic microbeads (Miltenyi Biotec). The purity of isolated CD14^+ ^cells was consistently > 90%, as determined by flow cytometry. To generate human MoDCs, CD14^+ ^monocytes were cultured in RPMI (Gibco BRL) supplemented with 10% FBS (Gibco BRL), 10 ng/ml IL-4 (Peprotech) and 50 ng/ml GMCSF (Peprotech) for 6 days, as we previously described [23]. The culture medium was refreshed on day 3, and cell differentiation was monitored using light microscopy. At day 6 of the culture, the purity of CD14^-^CD11c^+ ^cells was consistently > 95%, as determined by flow cytometry.

### Virus preparation, titration and infection

Human influenza virus A H1N1 (A/Hong Kong/54/98) was cultured in Madin-Darby canine kidney cells (American Type Culture Collection), as we described [24,25]. Virus titer was determined by daily observation of cytopathic effect, and the median tissue culture infective dose was calculated according to the Reed-Muench formula. Immature MoDCs on day 6 of the culture were washed before being infected with live H1N1 (strain HK/54/98) at the indicated multiplicity of infection (MOI). After 1 hr of viral adsorption, cells were washed with excess PBS to remove unadsorbed virus.

### Bacterial preparation, titration and stimulation

A clinical isolate of *Streptococcus pneumoniae *serotype 14 (strain 09M45278) recovered from blood sample of a patient treated in a regional hospital in Hong Kong in 2009 was used. The pneumococcal isolate was stored at -70°C. For recovery, an inoculated bead was directly streaked onto 5% horse blood agar and grown 14 hrs at 37°C, 5% CO_2. _The colony growths were passaged onto horse blood agars and grown for another 14 hrs at 37°C, 5% CO_2_. The colony growths from second pass were harvested by picking to pre-warmed PBS at 37°C. Bacterial number was measured by OD absorbance at 600 nm, and bacterial counts (CFU/ml) were confirmed by plating samples of 10-fold dilutions of bacteria on blood agar plates. *S. pneumoniae *was heat killed by incubating at 60°C for 1 hr as described [26]. Sterility was confirmed by subculture on blood agar plates. Prepared pneumococcus was kept at 4°C and used within one week. For every 1 × 10^6 ^immature or virus infected MoDCs, cells were washed and challenged with heat killed *S. pneumoniae *at 1 × 10^6^, 5 × 10^6 ^or 10 × 10^6 ^CFU. Pneumococcus was added to MoDCs cultured in 24-well plate. The plate was centrifuged to achieve synchronization of stimulation [27].

### Viral bacterial stimulation protocol

MoDCs were challenged with the combination of live influenza virus and heat killed pneumococcus either concurrently or successively. In the concurrent challenge, MoDCs were first infected with H1N1 at MOI 0.1 for 0 hr, and then immediately stimulated with heat killed pneumococcus at 1 × 10^6^, 5 × 10^6 ^or 10 × 10^6 ^CFU for 24 hr. The total incubation time at harvest was 24 hr. As a control, MoDCs were infected with H1N1 alone or treated with pneumococcus alone for 24 hr. In the successive challenge, MoDCs were first infected with H1N1 at MOI 0.1 for either 6 hr or 24 hr, and then treated with heat killed pneumococcus at 1 × 10^6^, 5 × 10^6 ^or 10 × 10^6 ^CFU for another 24 hr to make up a total incubation time at harvest of 30 hr or 48 hr respectively. As a control, for the 6 hr successive challenge group, MoDCs were infected with H1N1 alone for 30 hr, or treated with pneumococcus alone for 24 hr. For the 24 hr successive challenge group, MoDCs were infected with H1N1 alone for 48 hr, or treated with pneumococcus alone for 24 hr. In all the three groups of viral bacterial challenge, MoDCs were mock treated for 48 hr as negative control.

### Cell staining and flow cytometry

For surface staining, MoDCs were washed and then stained with APC conjugated anti-CD83 (HB15e), PE conjugated anti-CD86 (IT2.2; both from BioLegend) and FITC conjugated anti-HLA-DR, DP, DQ (Tu39; BD Pharmingen) together in one tube. Cells incubated with their relevant isotype controls were served as controls. To avoid non-specific staining, cells were incubated in PBS with 2 to 5% FBS during the staining process. MoDC death was determined by PI staining in a separate tube. MoDC apoptosis was determined using FITC Annexin V apoptosis detection kit I (BD Pharmingen) according to manufacturer's instructions. All data were acquired on BD FACSAria with FACS Diva (BD Biosciences) and analyzed using FlowJo software (TreeStar) as we described previously [28-30]. At least 15,000 gated events were acquired per sample.

### Quantification of cytokines by flowcytomix kits

The culture supernatant collected was assayed to determine cytokine concentration, including TNF-α, IL-6, IL-12, IFN-γ and IL-10 using FlowCytomix kits (Bender MedSystems) according to manufacturer's instructions.

### Statistical analysis

Data are expressed as means ± standard deviation (SD). Statistical analysis was performed by Repeated Measures ANOVA with Bonferroni multiple comparison test using Prism 4 (GraphPad Software). P value < 0.05 was considered significant.

## Results

### MoDCs response to influenza virus infection

**Figure 1:**
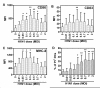
**Response of MoDCs to influenza virus**. MoDCs were infected with influenza virus H1N1 (HK/54/98) at the indicated MOI. 24 hr after infection, cells were harvested and the surface expressions of CD86, CD83 and MHC-II (A-C) were examined to determine phenotypic maturation. Cells were stained with PI to examine the percentage of dead cells after infection (D). Data (n = 4) represented mean ± SD of four individual experiments. Statistical significance compared to MOI 0 *p < 0.05, **p < 0.01 (ANOVA).

Influenza virus induces apoptosis of immune cells [24,31,32]. We first investigated the dose response of MoDCs to live influenza virus. Gating on the total MoDC population at 24 hr after infection, an infectious dose of MOI 0.05 induced CD86 and MHC-II expression significantly (Figure 1A, C). The percentage of PI^+ ^MoDCs significantly increased at MOI 0.3 and reached a plateau of almost 30% at MOI 1 (Figure 1D). Since influenza virus infection at MOI 0.1 induced MoDC maturation, yet without causing a considerable percentage of cell death, MOI 0.1 was chosen to be the infectious dose for the following experiments. As most viruses are poor at replication once being internalized by dendritic cells [33], we next investigated whether the influenza virus infection of MoDCs was productive or abortive. MoDCs were infected with H1N1 at MOI 0.1, no infectious virus was detected in the culture supernatant collected at 0 hr, 6 hr, and 24 hr post-infection, which indicated that the infection could not yield intact virions (data not shown).

### Successive challenge of influenza virus and pneumococcus induced greater MoDC apoptosis

**Figure 2:**
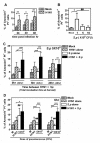
**MoDC apoptosis induced by successive challenge of influenza virus and pneumococcus**. (A) MoDCs were infected with H1N1 at MOI 0.1. The percentage of apoptotic cells was examined at 24 hr, 30 hr or 48 hr after virus infection. (B) MoDCs were treated with heat killed pneumococcus at 1 × 10^6^, 5 × 10^6 ^or 10 × 10^6 ^CFU. The percentage of apoptotic cells was examined at 24 hr after pneumococcal stimulation. (C-D) MoDCs were concurrently or successively challenged by both influenza virus and heat killed pneumococcus. (C) MoDCs were first infected with H1N1 at MOI 0.1 for 0 hr, and then immediately treated with 5 × 10^6 ^CFU heat killed pneumococcus for another 24 hr. The total incubation time at harvest was 24 hr. Alternatively, MoDCs were first infected with H1N1 at MOI 0.1 for 6 hr or 24 hr, and then treated with 5 × 10^6 ^CFU heat killed pneumococcus for another 24 hr to make up a total incubation time at harvest of 30 hr or 48 hr respectively. (D) MoDCs were infected with H1N1 at MOI 0.1 for 6 hr, and then treated with 1 × 10^6^, 5 × 10^6 ^or 10 × 10^6 ^CFU heat killed pneumococcus for another 24 hr, with a total incubation time at harvest was 30 hr. Data (n = 6) represented mean ± SD of five individual experiments. Please note that some groups of data were presented twice in different panels for easy interpretation. *p < 0.05, **p < 0.01, ***p < 0.001 (ANOVA) and ##p < 0.01 (Post test for trend).

To examine cell survival, MoDCs were treated with influenza virus, heat killed pneumococcus, or in combination of the two pathogens, and the cell apoptotic frequency was examined by Annexin V and PI staining. Influenza virus infection alone at MOI 0.1 mildly increased the percentage of apoptotic cells (Figure 2A). Heat killed pneumococcus at 1 × 10^6 ^CFU significantly induced cell apoptosis, but increase of dose to 5 × 10^6 ^CFU and above had no effect in enhancing cell apoptosis (Figure 2B). In the viral bacterial challenge, when MoDCs were treated with 5 × 10^6 ^CFU heat killed pneumococci at 0 h or 6 hr after viral infection, the percentage of apoptotic cell was significantly higher than that treated with virus alone or pneumococcus alone (Figure 2C). However, when MoDCs were treated with heat killed pneumococcus at 24 hr after viral infection, the percentage of apoptotic cell was only significantly higher than that treated with pneumococcus alone. These results indicated that the induction of greater cell apoptosis during the successive challenge was dependent on the timing between the administration of influenza virus and pneumococcus. Furthermore, the low dose of pneumococcal stimulation alone was effective to induce MoDC apoptosis, and there was a significant decreasing trend of cell apoptosis upon the increasing dose of pneumococcal stimulation (Figure 2B). However, this dose dependent effect was abolished when pneumococcal stimulation was preceded by influenza virus infection (Figure 2D, comparing the loss of significant trend of white column with black column).

### Successive challenge of influenza virus and pneumococcus caused a time related change in MoDC phenotype

**Figure 3:**
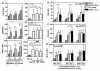
**MoDCs phenotype expression induced by successive challenge of influenza virus and pneumococcus**. (A) MoDCs were infected with H1N1 at MOI 0.1 or treated with heat killed pneumococcus at 1 × 10^6^, 5 × 10^6 ^or 10 × 10^6 ^CFU. Cell phenotypic maturation was examined at 24 hr, 30 hr or 48 hr after virus infection, or at 24 hr after pneumococcal stimulation. (B-D) MoDCs were concurrently or successively challenged by both influenza virus and heat killed pneumococcus. MoDCs were first infected with H1N1 at MOI 0.1 for 0 hr, and then immediately treated with 5 × 10^6 ^CFU heat killed pneumococcus for another 24 hr. The total incubation time at harvest was 24 hr. Alternatively, MoDCs were first infected with H1N1 at MOI 0.1 for 6 hr or 24 hr, and then treated with 5 × 10^6 ^CFU heat killed pneumococcus for another 24 hr to make up a total incubation time at harvest of 30 hr or 48 hr respectively. Data (n = 5-7) represented mean ± SD of six individual experiments. Please note that some groups of data were presented twice in different panels for easy interpretation. *p < 0.05, **p < 0.01, ***p < 0.001 (ANOVA) and ###p < 0.001 (Post test for trend).

Maturation is an important process for dendritic cell to exercise its function. Gating on the viable population of MoDCs after pathogen stimulation, MoDCs were phenotypically activated by either influenza virus or heat killed pneumococcus (Figure 3A). In viral bacterial challenge, when MoDCs were treated with 5 × 10^6 ^CFU heat killed pneumococci at 0 hr or 6 hr after virus infection, the expression of CD83 was comparable to that in pneumococcal stimulated MoDCs, but it was significantly higher than that in virus infected MoDCs (Figure 3B). This suggested that pneumococcus tended to be the major contributor for the up-regulation of CD83. When compared to the influenza virus infection alone, MoDCs became more mature by the secondary pneumococcal challenge. When MoDCs were treated with 5 × 10^6 ^CFU heat killed pneumococci at 6 hr after virus infection, the expression of CD86 was significantly higher than that infected with influenza virus alone (Figure 3C). However, in all the conditions of the viral bacterial challenge performed, MoDCs treated with both the two pathogens expressed comparable level of MHC-II to that infected with virus alone or treated with pneumococcus alone (Figure 3D). Furthermore increase in secondary pneumococcal dose after influenza virus infection did not change the expression of CD83, CD86 and MHC-II on MoDCs treated with both the two pathogen significantly (data not shown).

### Successive challenge of influenza virus and pneumococcus dysregulated MoDC cytokine production

Another important function of mature dendritic cell is to produce cytokines to regulate immune response against pathogens. We investigated the induction of pro-inflammatory cytokines: TNF-α, IL-6, IL-12 and IFN-γ, as well as anti-inflammatory cytokine: IL-10 by FlowCytomix Multiplex following pathogens stimulation.

**Figure 4:**
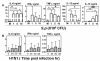
**MoDC cytokine profile in response to influenza virus or pneumococcus**. (A) MoDCs were treated with 1 × 10^6^, 5 × 10^6 ^or 10 × 10^6 ^CFU heat killed pneumococcus for 24 hr or (B) infected with H1N1 at MOI 0.1 for 0 hr, 24 hr, 30 hr or 48 hr. The concentration of cytokine in culture supernatant was determined. Data (n = 5-7) represented mean ± SD of six individual experiments. **p < 0.01 (ANOVA) and #p < 0.05, ###p < 0.001 (Post test for trend).

MoDCs produced IL-6, IFN-γ, TNF-α, IL-12 and IL10 after single heat killed pneumococcal challenge. Increase in pneumococcal dose from 1 × 10^6 ^to 10 × 10^6 ^CFU, MoDCs gradually produced more IL-10 with a significant increasing trend, and less TNF-α and IL-12 with a significant decreasing trend (Figure 4A). Influenza virus infection alone at MOI 0.1 mildly induced TNF-α, IL-6 and IFN-γ from MoDCs (Figure 4B). The secretions of IL-12 and IL-10 from virus infected MoDCs were lower than the detectable limitation (data not shown). The lack of IL-12 and IL-10 response was consistent with DNA microarray study that gene expression of IL-12p40 and IL-10 from human MoDCs was unchanged in response to influenza virus [34], and myeloid DCs produced no IL-10 upon TLR3 agonist polyIC stimulation [35].

**Figure 5:**
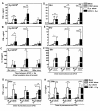
**MoDC pro-inflammatory cytokines in response to successive challenge of influenza virus and pneumococcus**. (A-H) MoDCs were concurrently or successively challenged by both influenza virus and heat killed pneumococcus. (A-C) MoDCs were infected with H1N1 at MOI 0.1 for 0 hr, 6 hr or 24 hr, and then treated with 5 × 10^6 ^CFU heat killed pneumococcus for another 24 hr to make up a total incubation time at harvest of 24 hr, 30 hr or 48 hr respectively. (D-F) MoDCs were infected with H1N1 at MOI 0.1 for 6 hr, and then treated with 1 × 10^6^, 5 × 10^6 ^or 10 × 10^6 ^CFU heat killed pneumococcus for another 24 hr, with a total incubation time at harvest was 30 hr (G-H) MoDCs were first infected with H1N1 at MOI 0.1 for 24 hr, and then treated with 1 × 10^6^, 5 × 10^6 ^or 10 × 10^6 ^CFU heat killed pneumococcus for another 24 hr, with a total incubation time at harvest was 48 hr. Data (n = 5-7) represented mean ± SD for six individual experiments. Please note that some groups of data were presented twice in different panels for easy interpretation. *p < 0.05, **p < 0.01, ***p < 0.001 (ANOVA), post test for trend between white columns #p < 0.05 and black columns ^^ p < 0.01.

In the viral bacterial challenge, the regulation of pro-inflammatory cytokine could be synergistic or non-synergistic. First, a synergistic production of TNF-α (Figure 5A), IFN-γ (Figure 5B) or IL-12 (Figure 5C) was observed when MoDCs were stimulated with heat killed pneumococcus at 6 hr, but not 0 hr nor 24 hr after virus infection. This demonstrated that the time between the consecutive viral bacterial challenges was a critical factor to regulate the inflammatory response. In the 6 hr successive challenge group, when the viral bacterial synergism occurred, increase in the dose of secondary pneumococcus promoted a stronger production of TNF-α (Figure 5D), IFN-γ (Figure 5E) or IL-12 (Figure 5F). These results indicated that successive challenge of influenza virus and pneumococcus induced a time and dose dependent synergism in the production of pro-inflammatory cytokines. Furthermore, in the regulation of TNF-α, increasing dose of heat killed pneumococcus alone from 1 × 10^6 ^to 10 × 10^6 ^CFU resulted in a significant decreasing trend of TNF-α production. However, this decreasing trend was reversed to that of a significant increasing trend of TNF-α production when the pneumococcal stimulation was preceded by influenza virus infection (Figure 5D, compare the significant trend of white column with black column). Taken together, these data suggested that successive challenge of influenza virus and pneumococcus on MoDCs generally promoted inflammatory response.

Second, in the 24 hr successive challenge group, when the viral bacterial synergism was absent, the dose of pneumococcus became another important factor to regulate the inflammatory response. Beginning with a low dose (1 × 10^6 ^CFU) of heat killed pneumococcal stimulation after virus infection, MoDCs produced significantly lower amount of TNF-α (Figure 5G) and IL-6 (Figure 5H) than that treated with pneumococcus alone. While the dose of secondary pneumococcus increased to 5 × 10^6 ^and 10 × 10^6 ^CFU, the production of TNF-α increased and eventually became significantly higher than that treated with pneumococcus alone (Figure 5G). Similarly the reduced production of IL-6 was rescued with the increasing dose of pneumococcus (Figure 5H). This demonstrated a competition between influenza virus and pneumococcus during the successive challenge, with the virus infection tended to lower pro-inflammatory cytokines secretion, whereas the increasing pneumococcal stimulation tended to promote inflammatory response.

**Figure 6:**
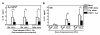
**MoDC anti-inflammatory cytokine in response to successive challenge of influenza virus and pneumococcus**. MoDCs were concurrently or successively challenged by both influenza virus and heat killed pneumococcus. (A) MoDCs were first infected with H1N1 at MOI 0.1 for 0 hr, 6 hr or 24 hr, and then treated with 5 × 10^6 ^CFU heat killed pneumococcus for another 24 hr to make up a total incubation time at harvest of 24 hr, 30 hr or 48 hr respectively. (B) MoDCs were first infected with H1N1 at MOI 0.1 for 6 hr, and then treated with 1 × 10^6^, 5 × 10^6 ^or 10 × 10^6 ^CFU heat killed pneumococcus for another 24 hr, with a total incubation time at harvest was 30 hr. Data (n = 5-7) represented mean ± SD for six individual experiments. Please note that some groups of data were presented twice in different panels for easy interpretation. *p < 0.05 (ANOVA).

In the regulation of anti-inflammatory cytokine, a negative antagonistic interaction of influenza virus and pneumococcus was observed in the induction of IL-10. While MoDCs produced IL-10 significantly after heat killed pneumococcal stimulation at 5 × 10^6 ^CFU, the IL-10 induced by pneumococcus alone was significantly inhibited when pneumococcal stimulation was preceded by influenza virus infection (Figure 6A). The inhibition of IL-10 was independent of the time intervals between the successive viral and pneumococcal challenge (Figure 6A). Furthermore, with the dose of secondary pneumococcus increase to 10 × 10^6 ^CFU, the inhibition of IL-10 by prior influenza virus infection remained significant (Figure 6B).

Taken together, the interaction of influenza virus and pneumococcus in the regulation of MoDC cytokine production was complex with both time and dose dependency. There were synergistic induction of pro-inflammatory cytokines and significant suppression of anti-inflammatory cytokine, which was dependent on the different permutations of the time intervals between the challenge of the two pathogens and pneumococcal dose.

## Discussion

Influenza virus co-infection with pneumococcus often leads to severe complications [1,2,14,15]. The present study addressed the dysregulation of MoDCs to viral bacterial challenge and provided direct descriptions of how human myeloid DCs respond to the two human pathogens. The major finding was that successive challenge of influenza virus and pneumococcus resulted in time and dose dependent changes in MoDC functions which promoted inflammation.

Viral bacterial co-infection accounts for substantial illness and death in seasonal and pandemic influenza [3-13]. Such co-infection can be either mixed viral bacterial infection with two pathogens at the beginning or sequential infection with pneumococcus usually comes in 5 to 7 days after viral infection. We mimic the viral bacterial co-infection and treat MoDCs with three different doses of pneumococcus at three different time points after the initial influenza virus infection. This allows us to capture the dynamic changes of MoDC response to these nine different permutations of time and dose related challenging.

First we demonstrated a general characteristic of MoDCs during the successive viral bacterial challenging, in which comprised of up-regulation of pro-inflammatory cytokines and down-regulation of anti-inflammatory cytokine. This finding suggested the possible contribution of dendritic cell to the inflammatory response in co-infection. Successive challenge of influenza and pneumococcus enhanced pro-inflammatory cytokines from MoDCs, which could potentially explain the human autopsy material from victim in 1918 Spanish flu showed the lung pathology of bacterial bronchopneumonia with intense inflammation [36,37], and the animal data with elevated lung inflammatory cytokines, as well as greater destruction of lung architecture during the co-infection [18,38].

Second, we demonstrated successive challenge of influenza virus and pneumococcus increased MoDCs apoptosis. The underlying mechanism of the increased MoDC apoptosis is not clear. A previous study indicated that autocrine IL-10 from BMDCs upon heat killed pneumococcal stimulation was crucial for delaying the onset of BMDC apoptosis [26]. Here, we also found that a high dose of heat killed pneumococcus alone induced IL-10 from MoDCs (Figure 4A) and did not cause significant cell apoptosis (Figure 2B), while prior influenza virus infection of MoDCs inhibited the IL-10 induced by pneumococcus (Figure 6) and increased cell apoptosis (Figure 2C). These results suggest that the reduced survival of MoDCs may be related to the decreased IL-10 response in MoDCs during the successive challenge of virus and pneumococcus.

Apoptotic DCs have low efficiency for T cell priming and are ineffective at inducing immunity. The enhanced MoDC apoptosis from successive challenge might enhance disease severity of viral bacterial co-infection. For the 6 hr successive challenge group, there were both marked up-regulation of cell apoptosis and pro-inflammatory cytokine production. Apoptosis could be a strategy for the resolution of inflammatory response [39]. Alveolar neutrophil apoptosis has been suggested to help down-regulating lung inflammation in patients with community acquired pneumonia [40]. However, there is no report describing the possible relationship between inflammation and apoptosis on dendritic cells. On the other hand, acute inflammatory response could lead to cell apoptosis [41], and the dying cells might in turn alert immune system to become activated [42]. In vivo, TNF-α was reported to block apoptotic cell clearance by alveolar macrophages, and exacerbated inflammatory response in the lung [43]. Our findings suggested that inflammation and cell death might interact with each other and contributed to the severe lung injury in co-infection.

Third, we found that influenza virus and pneumococcus synergistically or non-synergistically dysregulated MoDC cytokine response. In the synergistic regulation, virus and pneumococcus worked together to alter MoDC functions. This was observed when cell were stimulated with high dose of pneumococcus at 6 hr, but not 0 h nor 24 hr after the influenza virus infection. Our data clearly revealed that the viral bacterial synergism might only occur when secondary pneumococcus was introduced during the critical time period. These findings are consistent with mouse study showing a time and dose related synergistic lethality of influenza virus and pneumococcus [19], in which the greatest mortality of sequential infection occured when secondary pneumococal infection comes at 7 days, but not 0 to 3 days nor 14 to 21 days after influenza virus infection. The window period for the second challenge will of course be different between in vitro and in vivo study. The critical window period in altering MoDC functional changes suggested that there could be different critical time window for mixed and sequential viral bacterial infection. The role of the time interval between influenza virus infection and pneumococcal challenge in dysregulating MoDC functions is consistent with the suggested importance of the timing in two successive infections on the magnitude of inflammation [16].

On the other hand, in certain permutation of time intervals and pneumococcal doses, when the viral bacterial synergism was absent, the dose of pneumococcus was the determining factor to change MoDC response. For instance, with a low dose of pneumococcus, the preceding virus infection appeared to be determinative in skewing the MoDC cytokine production towards suppression of TNF-α, IL-6 and IL-10 as stimulated by the bacteria. In contrast, with the increasing dose of pneumococcus, the bacteria gradually dominated its effect over influenza virus, and turned the MoDC cytokine production towards up-regulation of pro-inflammatory cytokines: TNF-α, IFN-γ, IL-12 and IL-6.

Collectively, our data indicated that MoDC response to pathogen challenge is very plastic, dependent on the combination of time interval between the challenges of the two pathogens as well as the dose of the pathogens. This agrees with the study showing that human MoDCs can elicit both shared core and tailored pathogen specific immune response [34]. However, the present findings are limited to in vitro study, and future in vivo experiments would help better understand how the innate immune cells may differentially respond to the co-infection. In additions, the underlying mechanisms that integrate the different signals delivered by the influenza virus infection and pneumococcal challenge resulting in the observed cell response remain to be elucidated. Furthermore, the infective environment in which DCs are conditioned can influence the way they control and regulate subsequent T cell response [44]. As our findings demonstrated that successive challenge of MoDCs with influenza virus and pneumococcus up-regulated IL-12 and IFN-γ, which could skew developing immune responses toward Th1, we shall further investigate the regulation of adaptive immunity to co-infection.

## Conclusions

In summary, successive challenge of influenza virus and pneumococcus favour the production of pro-inflammatory cytokines and suppression of anti-inflammatory cytokine from human MoDCs, which are both time interval and pneumococcal dose dependent, reflecting the plasticity of dendritic cell to combination of pathogens. Our study contributes to the understanding of the underlying pathogenesis of severe bacterial infection associated with influenza.

## Competing interests

The authors declare that they have no competing interests.

## Authors' contributions

YLL, WT, YW designed the study. YW performed experiments. KHC prepared pneumococcus. YLL, WT, YW, HWM, MTL analyzed data and discussed findings. YLL, WT, PLH, YW, HWM wrote manuscript. All authors read and approved the final manuscript.

## Pre-publication history

The pre-publication history for this paper can be accessed here:

http://www.biomedcentral.com/1471-2334/11/201/prepub
